# Color Measurement of Tea Leaves at Different Drying Periods Using Hyperspectral Imaging Technique

**DOI:** 10.1371/journal.pone.0113422

**Published:** 2014-12-29

**Authors:** Chuanqi Xie, Xiaoli Li, Yongni Shao, Yong He

**Affiliations:** 1 College of Biosystems Engineering and Food Science, Zhejiang University, Hangzhou, China; 2 Agricultural and Biological Engineering Department, University of Florida, Gainesville, Florida, United States of America; 3 Key Laboratory of Equipment and Informatization in Environment Controlled Agriculture, Ministry of Agriculture, Hangzhou, China; CSIRO, Australia

## Abstract

This study investigated the feasibility of using hyperspectral imaging technique for nondestructive measurement of color components (ΔL*, Δa* and Δb*) and classify tea leaves during different drying periods. Hyperspectral images of tea leaves at five drying periods were acquired in the spectral region of 380–1030 nm. The three color features were measured by the colorimeter. Different preprocessing algorithms were applied to select the best one in accordance with the prediction results of partial least squares regression (PLSR) models. Competitive adaptive reweighted sampling (CARS) and successive projections algorithm (SPA) were used to identify the effective wavelengths, respectively. Different models (least squares-support vector machine [LS-SVM], PLSR, principal components regression [PCR] and multiple linear regression [MLR]) were established to predict the three color components, respectively. SPA-LS-SVM model performed excellently with the correlation coefficient (*r_p_*) of 0.929 for ΔL*, 0.849 for Δa*and 0.917 for Δb*, respectively. LS-SVM model was built for the classification of different tea leaves. The correct classification rates (CCRs) ranged from 89.29% to 100% in the calibration set and from 71.43% to 100% in the prediction set, respectively. The total classification results were 96.43% in the calibration set and 85.71% in the prediction set. The result showed that hyperspectral imaging technique could be used as an objective and nondestructive method to determine color features and classify tea leaves at different drying periods.

## Introduction

Tea is welcome by many people because of its healthy function. For example, it can prevent cancer and cardiovascular disease and cure chronic gastritis [1], [2]. Tea processing procedure, which is composed of a series of physical and chemical reactions, can affect tea's quality directly [3]. However, the change of color values (ΔL*, Δa* and Δb*) of tea leaves play significant roles in tea processing procedure. Therefore, studying color parameters of tea leaves during drying periods can finally improve tea's quality.

**Figure 1:**
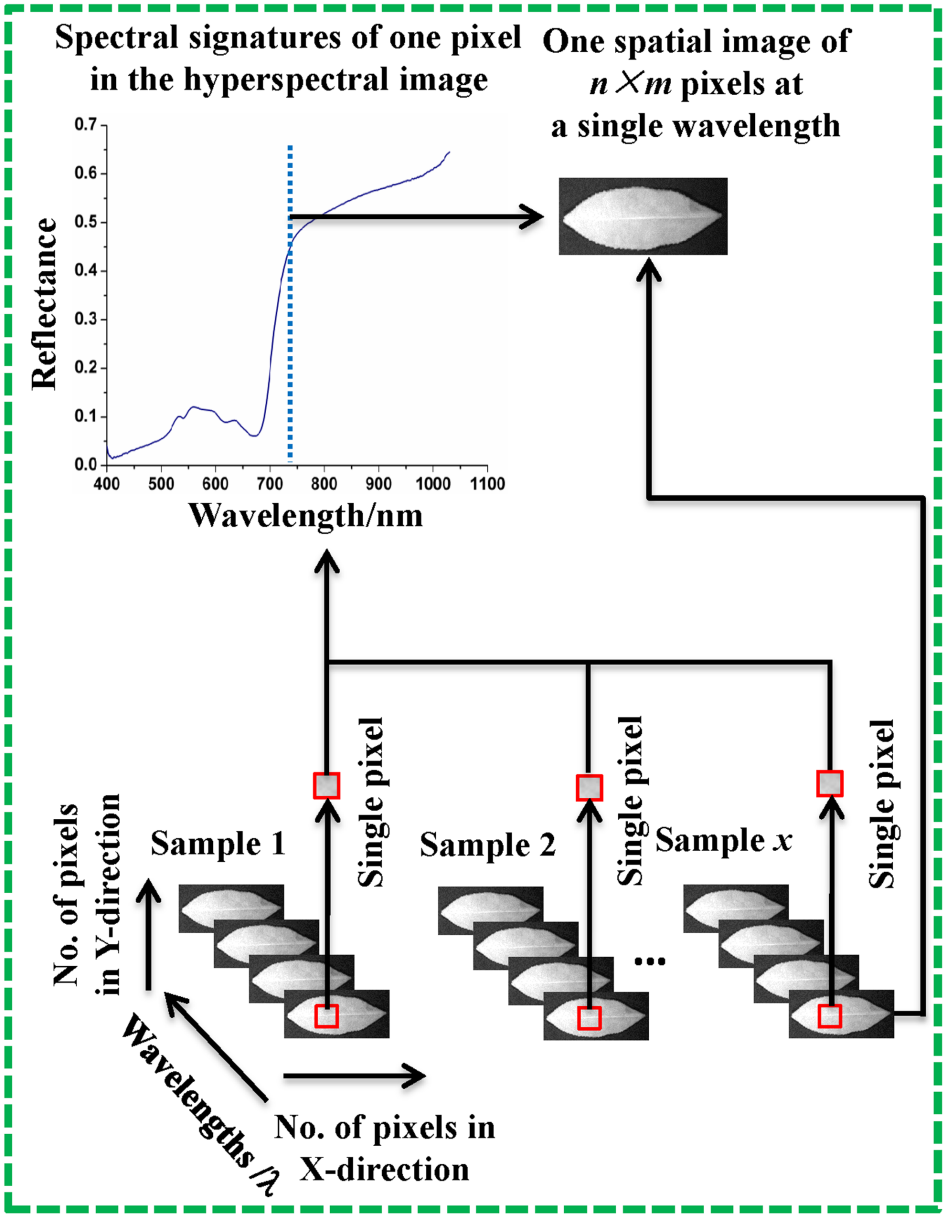
Hyperspectral imaging.

Hyperspectral imaging technique, which integrates both spectral and imaging techniques, has been widely applied in many fields [4], [5], [6]. A spatial picture can be generated when the sample is scanned by the hyperspectral imaging system. The spatial picture (hyperspectral cube) consists of a series of images at different wavelength, and each pixel has both spectroscopic and spatial information. The schematic hyperspectral imaging system can be seen in Fig. 1. In accordance with the previous studies, hyperspectral imaging technique is very efficient for knowing the process when the samples changes with time [7], [8].

Hyperspectral technique has many advantages, such as nondestructive, rapid and simple operation, accurate, low cost, and can be applied in on-line detection. At present, hyperspectral technique has already been used to detect color parameters in many studies [9], [10], [11], [12], [13]. The change of color parameters of tea leaves is very important in the tea processing procedure. However, to measure the color parameters of tea leaves at different drying periods using hyperspectral imaging technique has not been found.

The goals of this work were: (1) to find the quantitative relationships between the spectral reflectance information and color parameters of tea leaves at different drying periods; (2) to obtain effective wavelengths which are useful for the determination of color values; (3) to compare the predictive ability of different calibration models; (4) to develop an algorithm for the determination of color values of tea leaves.

## Materials and Methods

### Hyperspectral imaging system

**Figure 2:**
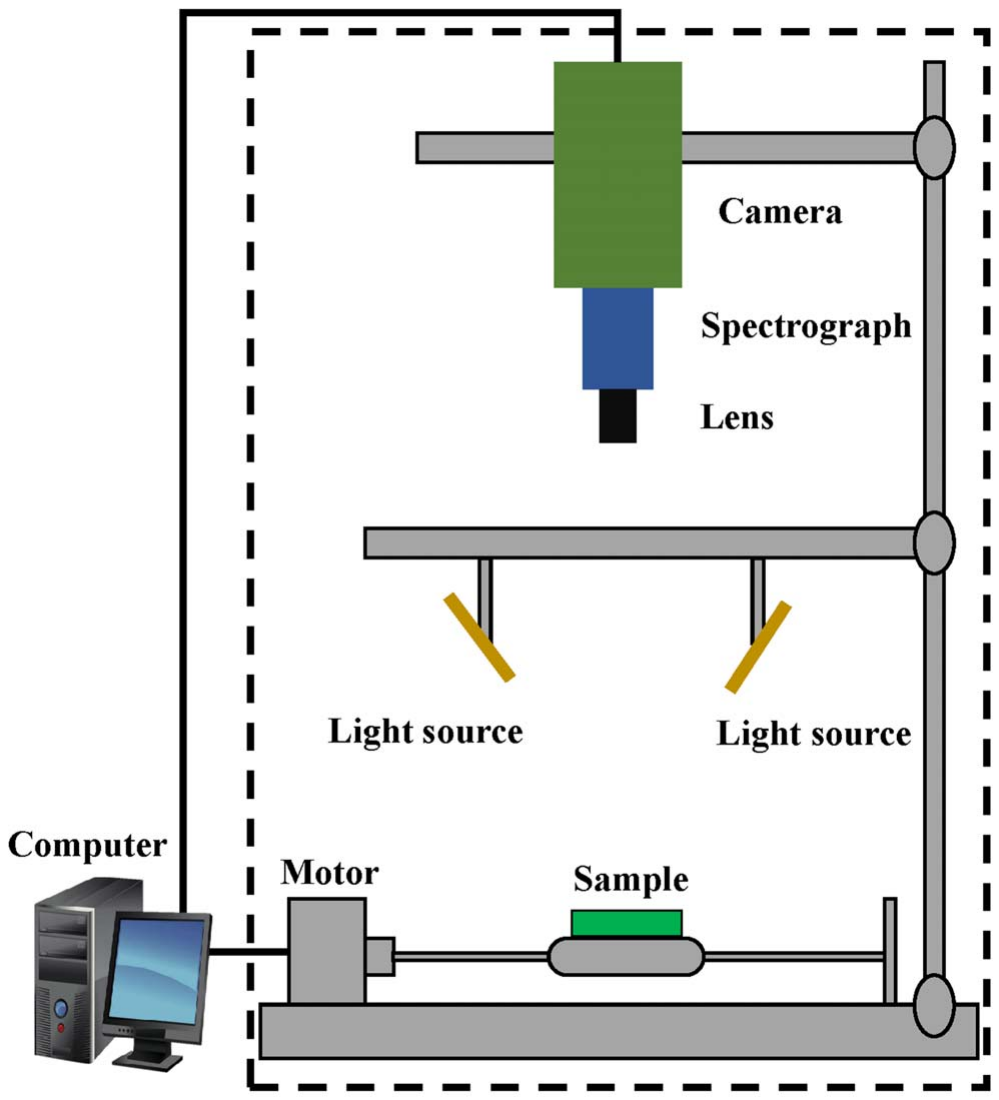
Schematic diagram of the hyperspectral imaging system.

A visible and near infrared (VIS-NIR) hyperspectral imaging system covering the spectral wavelengths of 380–1030 nm was used in this study (as shown in Fig. 2). The system includes a lens (OLE-23), an imaging spectrograph (V10E-QE, Specim, Finland), a CCD camera (C8484-05, Hamamatsu City, Japan), two light sources (Oriel Instruments, Irvine, USA) provided by two 150W quartz tungsten halogen lamps, a conveyer belt operated by a stepper motor (IRCP0076, Isuzu Optics Corp., Taiwan, China), and a computer operating the spectral image system V10E software (Isuzu Optics Corp., Taiwan, China). The area CCD array detector of the camera has 672×512 (spatial × spectral) pixels, and the spectral resolution is 2.8 nm. The system scans the samples line by line, and the reflected light was dispersed by the spectrograph and captured by the area CCD array detector in spatial-spectral(*x*×*λ*) axes. The ENVI 4.7 (Research system Inc., Boulder, Co., USA), Unscrambler V9.7 (CAMO Process AS, Oslo, Norway) and Matlab R2009a (The Math Works, Inc., Natick, MA, USA) software were used in this study.

### Samples preparation and flow chart

Three different cultivars of tea leaves including Biyun, Longjing-43 and Zhongcha-302 were used in this study. All of them were very famous tea in China. The number of each cultivar was fourteen. All leaves were picked from the green house, which is located at Zhejiang University, Hangzhou (120.2E, 30.3N), China. First of all, hyperspectral images of forty-two fresh tea leaves were acquired, and then three color parameters (ΔL*, Δa* and Δb*) of these fresh tea leaves were measured using the colorimeter (Konica Minolta, SC-80C, Japan). These leaves were then dried in a drying oven (GHD-9070A, JingHong, Shanghai, China) at 80°C for four minutes. After being cooled in a glass desiccator, they were imaged and color measured again. The same operation was run three more times with the same drying temperature (the third operation for six minutes, the fourth operation for eight minutes and the fifth operation for ten minutes).

**Figure 3:**
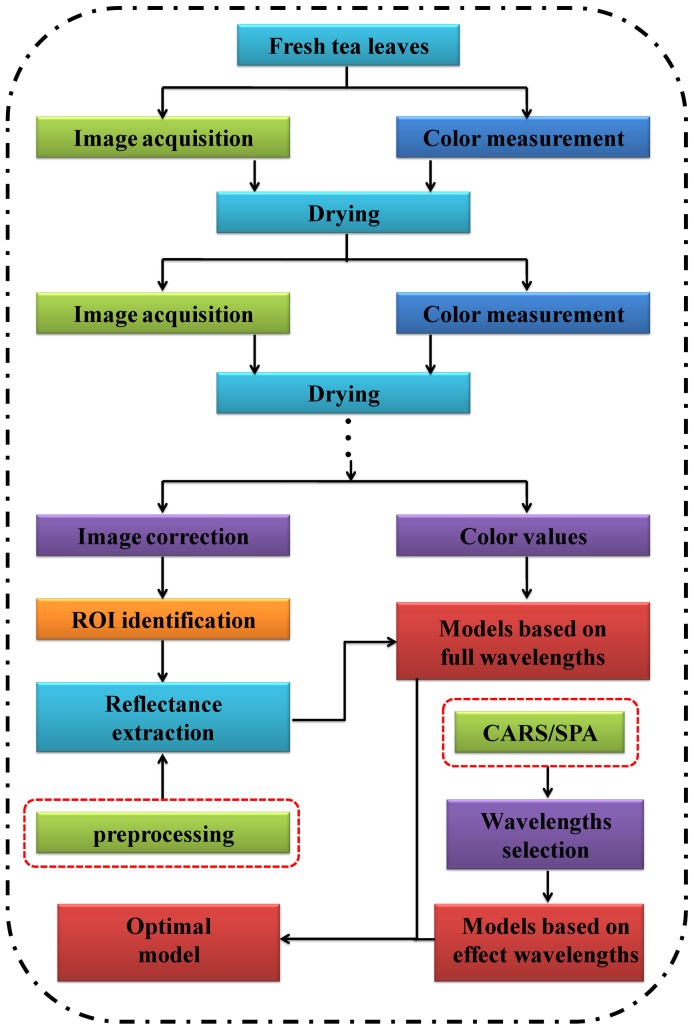
Main steps of this study.

The main steps of this study are illustrated in Fig. 3. All raw hyperspectral images were acquired by the hyperspectral imaging system in the wavelengths of 380 to 1030 nm. Simultaneously, color values were determined by the colorimeter. The raw hyperspectral images were then corrected by the dark and white reference images. Spectral reflectance values of all pixels from the ROI (30×30 pixels) of each sample were extracted and averaged as one variable. Thus, a total of 210 *X* variables were obtained and used to represent the spectral data of all samples. Then, these samples were divided into two sets at a ratio of 2∶1 (2/3 for calibration and 1/3 for prediction). Nine different pre-processing methods were used to improve the predictive ability. In order to optimize calibration model, two effective wavelengths selection methods including competitive adaptive reweighted sampling (CARS) and successive projections algorithm (SPA) were used to select the key wavelengths. The optimal calibration model was determined according to the values of *r_c_, r_p_*, *RMSEC* and *RMSEP*.

### Image acquisition and correction

The exposure time was 0.07 s, the moving speed was 2.6 mm/sec, and the vertical distance between the lens and sample was 36.0 cm. Each leaf was then placed on the conveyer belt to be scanned using the hyperspectral imaging system. Then, one hyperspectral image (hyperspectral cube) for each sample was obtained covering the spectral wavelengths of 380 to 1030 nm. The dimensions of the hyperspectral cube were 512 bands in the *λ* dimension and 672 pixels in the *y* dimension. When raw hyperspectral images were created, they should be corrected with dark and white reference images based on equation (1). The dark reference image with the reflectance factor of about 0% was obtained by covering the lens with the cap and turning off the light. The white reference image with the reflectance factor of about 99% was obtained from a white Teflon board (CAL-tile200, 200 mm×25 mm×10 mm).
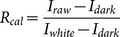
(1)


Where *R_cal_* is the corrected hyperspectral image, *I_raw_* is the raw hyperspectral image, *I_dark_* is the dark reference image, and *I_white_* is the white reference image.

### Models and evaluation index

Partial least squares (PLS) is an effective method which has been widely used for establishing calibration models in many spectral studies [14], [15], [16], [17], [18]. This algorithm is very efficient when predicting many different measured variables that are collinear. The spectral information is projected onto a small number of latent variables (LVs) for compressing the original spectral data [19]. The predicted result is achieved by extracting a set of orthogonal factors which carry most of the useful information for predicting [20]. Principal components regression (PCR), which can not only compress the high dimension of the raw variables effectively but also speed up the calculation by ignoring the minor components, has also been widely applied in many studies [21], [22]. The PCR algorithm can effectively overcome multi-collinearity problem which may result in instability for the predicted result. Multiple linear regression (MLR) is a common method used to establish models due to its features, such as being simple and easy interpretation. Though it has been used in many studies [23], [24], it fails when the number of the sample is fewer than that of the input variables [25]. In this study, the number of the samples in calibration set was fewer than that of the full spectral variables. Thus, MLR model was only established based on the selected wavelengths suggested by CARS and SPA, respectively. PLS, PCR and MLR models were operated by Unscrambler V9.7 software for the determination of the three color parameters. Least Squares-Support Vector Machine (LS-SVM), which is a simplification of traditional Support Vector Machine (SVM), is capable of handle both linear and nonlinear multivariate problems in a fast way [26]. The advantage of this algorithm is that a linear set of equations instead of a quadratic programming (QP) problem were used to obtain support vectors (SV). This method has also been widely used in many fields [27], [28]. In this study, LS-SVM was used to build model for predicting color parameters and classify tea leaves during different drying periods. The calculation was carried out by the free LS-SVM toolbox (LS-SVM v1.5, Suykens, Leuven, Belgium) in Matlab R2009a.

The performance of the models was evaluated in accordance with the values of the correlation coefficient (*r_c_ and r_p_*), root mean square error of calibration (*RMSEC*) and root mean square error of prediction (*RMSEP*) [29]. A good model should be of high values of *r_c_ and r_p_*, low values of *RMSEC* and *RMSEP*, and small difference between *RMSEC* and *RMSEP*. A large difference between the values of *RMSEC* and *RMSEP* indicates that the model is over-fitting [30].

### Key wavelengths selection

For the purpose of improving the performance of the predictive ability and reducing the influence of redundant information between contiguous wavelengths in the whole spectrum, selection of effective wavelengths is a very significant operation in spectral studies [31]. According to the previous studies, the selected wavelengths can be equally or even more effective than the whole spectral wavelengths [32]. The CARS was firstly used to select effective wavelengths from the full spectral wavelengths in this study. The CARS selects the key wavelengths based on the principle of “survival of the fittest” [33]. It abandons the wavelengths which are of small regression coefficients by exponentially decreasing function (EDF). The main procedures of each sampling run can be described as follows: (a) model sampling based on Monte Carlo (MC) principle; (b) wavelengths selection using EDF; (c) competitive wavelengths selection based on adaptive reweighted sampling (ARS); (d) evaluation of the subset by cross validation. Finally, those wavelengths which contain little or no useful information are eliminated while effective wavelengths are retained [34], [35]. The SPA, which is also a robust method for the selection of key wavelengths, was then used to select effective wavelengths. This algorithm can solve the collinear problem by selecting optimal wavelengths with minimal redundancy, and use a projection operation in a vector space for selecting key wavelengths [36], [37]. Both of the two wavelengths selection algorithms were operated in Matlab R2009a. Finally, the raw spectral data were consequently reduced into a matrix with a dimension of *m×n* (*m* was the number of the samples, and *n* was the number of the selected wavelengths).

### Measurement of color values (ΔL*, Δa* and Δb*)

In tea processing procedure, color parameters of tea leaves play vital roles for the reason that they can not only directly determine tea's quality, but also reflect the quality. Therefore, it is crucial to acquire the color parameters of tea leaves at different drying periods. In this study, three color parameters (ΔL*, Δa* and Δb*) were measured using the colorimeter. Before color measurement, the colorimeter should be firstly calibrated by a standard white calibration plate. The parameter ΔL* is the lightness or luminance component. The other two parameters (Δa* and Δb*), which range from −120 to 120, are the two chromatic components. The parameters ΔL*, Δa* and Δb* represent color changes from dark to brightness, green to red and blue to yellow, respectively.

## Results and Discussion

### Statistics of color parameters

A total of 210 tea leaves at five different drying periods (fresh, drying for 4 min, drying for 6 min, drying for 8 min and drying for 10 min) were studied. They were divided into the calibration set and the prediction set at a ratio of 2∶1. That is one sample was picked out from every three ones consecutively, which resulted in 140 samples for the calibration set (Biyun: 46, Longjing-43: 47, Zhongcha-302: 47) and 70 ones for the prediction set (Biyun: 24, Longjing-43: 23, Zhongcha-302: 23). The detailed statistical values of each set are shown in Table 1.

**Table 1:** Reference values of color (ΔL*, Δa* and Δb*) of tea leaves in calibration and prediction sets.

Statistics	Calibration			Prediction		
	ΔL*	Δa*	Δb*	ΔL*	Δa*	Δb*
Minimum	−69.78	−9.66	7.56	−69.96	−9.27	8.87
Maximum	−55.59	1.81	30.43	−56.35	2.11	31.84
Mean	−62.77	−5.85	16.85	−62.78	−5.74	17.05
Standard Deviation	3.13	1.95	5.33	3.20	2.14	5.40

### Preprocessing results

In order to obtain useful spectral information and improve the predictive ability, wavelengths at the beginning with some noise were rejected, resulting in the wavelengths of 400 to 1030 nm were studied. Then, nine different preprocessing algorithms were used to evaluate the optimal one in terms of the values of *r_c_, r_p_*, *RMSEC* and *RMSEP* of PLS model. The results can be seen in Table 2. Based on the evaluation standards, raw data performed best with the highest values of *r_p_* (0.925 for ΔL* and 0.930 for Δb*, respectively). Though the PLS model based on MSC preprocessing method obtained the result with the highest values of *r_c_* (0.949) and *r_p_* (0.799) for Δa*, the lowest value of *RMSEC* (0.611) and the second lowest value of *RMSEP* (1.276), it did not performed well due to the big gap between the values of *r_c_* and *r_p_*. Thus, raw data was used for further study.

**Table 2:** Performance of models in calibration and prediction for predicting color (ΔL*, Δa* and Δb*) using different preprocessing methods.

Preprocessing	Calibration						Prediction						No.^h^
	ΔL*		Δa*		Δb*		ΔL*		Δa*		Δb*		
	*r_c_*	*RMSEC*	*r_c_*	*RMSEC*	*r_c_*	*RMSEC*	*r_p_*	*RMSEP*	*r_p_*	*RMSEP*	*r_p_*	*RMSEP*	
Raw	0.911	1.286	0.837	1.064	0.917	2.118	0.925	1.205	0.793	1.295	0.930	1.979	5/9/8
MAS^a^	0.910	1.288	0.898	0.855	0.918	2.107	0.925	1.204	0.781	1.327	0.928	1.991	5/13/9
SGS^b^	0.909	1.296	0.910	0.805	0.914	2.156	0.925	1.204	0.782	1.324	0.923	2.061	5/14/8
MFS^c^	0.910	1.289	0.896	0.863	0.913	2.166	0.925	1.207	0.780	1.328	0.925	2.045	5/13/8
GFS^d^	0.910	1.290	0.834	1.071	0.917	2.126	0.925	1.209	0.785	1.317	0.928	1.991	5/9/8
Normalize	0.912	1.276	0.820	1.113	0.917	2.117	0.925	1.209	0.793	1.294	0.927	2.011	4/7/7
MSC^e^	0.898	1.368	0.949	0.611	0.924	2.038	0.906	1.342	0.799	1.276	0.908	2.253	5/15/8
SGD^f^	0.947	1.004	0.775	1.229	0.961	1.472	0.914	1.289	0.618	1.670	0.904	2.293	7/4/8
Baseline	0.892	1.410	0.863	0.981	0.922	2.058	0.902	1.369	0.791	1.301	0.925	2.045	4/10/9
SNV^g^	0.898	1.367	0.942	0.652	0.924	2.037	0.906	1.342	0.804	1.263	0.909	2.236	5/14/8

a Moving average smoothing;

b Savitzky-Golay smoothing;

c Median filter smoothing;

d Gaussian filter smoothing;

e Multiplicative scatter correction;

f Savitzky-Golay derivatives;

g Standard normal variate;

h Number of latent variables of ΔL*/Number of latent variables of **Δ**a*/Number of latent variables of **Δ**b*.

### Effective wavelengths

In order to obtain the optimal model with the robust predictive ability and a small number of input variable, two wavelengths selection methods (CARS and SPA) were conducted to determine the most effective wavelengths in this study, respectively. As a result, forty-eight (ΔL*), thirty-four (Δa*) and twenty-six (Δb*) wavelengths were identified by CARS, respectively; seven (ΔL*), six (Δa*) and eleven (Δb*) wavelengths were selected by SPA, respectively. Compared with the number of full spectral wavebands, those of the selected wavelengths recommended by CARS only account for 9.68%, 6.85% and 5.24%, respectively. The numbers of effective wavelengths suggested by SPA were only 1.41%, 1.21% and 2.22% of that of the full wavebands, respectively. These selected wavelengths (as shown in Table 3) were then used to replace the whole spectral wavelengths for the determination of color values. A total of four different calibration models (LS-SVM, PLSR, PCR and MLR) were established based on the selected wavelengths, respectively. These selected wavelengths not only simplify the calibration model and speed up the calculation but also improve the accuracy and robustness of the predictive ability.

**Table 3:** Effective wavelengths recommended by CARS and SPA, respectively.

Methods	Type	Number	Effected wavelengths/nm
CARS	ΔL*	48	410, 413, 416, 420, 425, 429, 444, 448, 531, 539, 543, 544, 545, 638, 639, 677, 767, 869, 874, 884, 892, 922, 925, 929, 934, 937, 945, 958, 965, 969, 971, 973, 975, 977, 978, 981, 982, 985, 987, 998, 999, 1001, 1006, 1007, 1013, 1015, 1018, 1027
CARS	Δa*	34	407, 428, 429, 515, 517, 518, 519, 540, 543, 544, 548, 586, 588, 590, 610, 611, 613, 614, 615, 616, 676, 693, 724, 741, 922, 924, 950, 965, 971, 985, 986, 1014, 1017, 1021
CARS	Δb*	26	461, 462, 543, 545, 584, 585, 608, 609, 610, 700, 711, 729, 753, 846, 848, 965, 969, 974, 975, 977, 987, 989, 990, 1009, 1015, 1018
SPA	ΔL*	7	457, 540, 649, 735, 761, 874, 1017
SPA	Δa*	6	540, 608, 676, 690, 985, 1017
SPA	Δb*	11	404, 408, 414, 416, 418, 444, 540, 648, 770, 866, 971

### Predicted results

**Figure 4:**
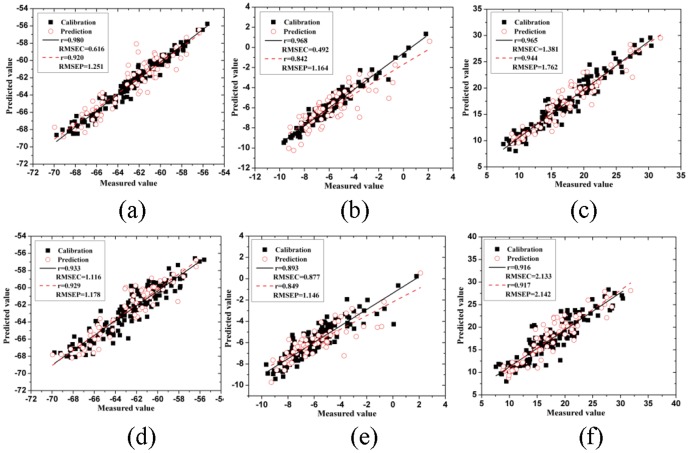
Measured vs. predicted values of calibration and prediction by CARS-LS-SVM and SPA-LS-SVM models, respectively. (a): CARS-LS-SVM-ΔL*; (b): CARS-LS-SVM-Δa*; (c): CARS-LS-SVM-Δb*; (d): SPA-LS-SVM-ΔL*; e): SPA-LS-SVM-Δa*; (f): SPA-LS-SVM-Δb*.

The predicted results can be seen in Table 4. On the basis of the evaluation index, CARS-MLR model performed perfectly with a satisfying result for ΔL* (*r_c_* = 0.963, *r_p_* = 0.931, *RMSEC* = 0.838 and *RMSEP* = 1.160). For Δa*, CARS-LS-SVM model obtained the best result with the *r_c_* of 0.968, *r_p_* of 0.842, *RMSEC* of 0.492 and *RMSEP* of 1.164, respectively. For Δb*, it was also the CARS-LS-SVM model that performed excellently with the *r_c_* of 0.965, *r_p_* of 0.944, *RMSEC* of 1.381 and *RMSEP* of 1.762, respectively. The number of input variables for these three models was 48, 34 and 26, respectively. Among the four models established based on SPA, SPA-LS-SVM model performed best with the highest values of *r_c_* (0.933 for ΔL*, 0.893 for Δa* and 0.916 for Δb*, respectively) and *r_p_* (0.929 for ΔL*, 0.849 for Δa* and 0.917 for Δb*, respectively), the lowest values of *RMSEC* (1.116 for ΔL*, 0.877 for Δa* and 2.133 for Δb*, respectively) and *RMSEP* (1.178 for ΔL*, 1.146 for Δa* and 2.142 for Δb*, respectively). From the results, it could be seen that LS-SVM model based on the selected wavelengths performed better than other models. Though there was a little decrement of the values of *r_c_* and *r_p_*, increment of the values of *RMSEC* and *RMSEP* for those models established based on SPA, the input variables were fewer compared with the models which were established based on CARS, respectively. The fewer input variables demonstrated that CARS and SPA can improve the performance of the predicted ability for the determination of color parameters. Thus, these selected wavelengths were more efficient than the whole wavelengths. It may because that the whole spectral wavelengths contained more redundant information which affects the performance of the predicted results. The predicted results of CARS-LS-SVM and SPA-LS-SVM models were shown in Fig. 4 (a–f), respectively. It could be found that the plots in calibration and prediction sets were distributed near the ideal lines, indicating that the performance of these models were good. It demonstrated that hyperspectral imaging technique could be used to determine the color parameters of tea leaves, and both CARS and SPA methods could remove uninformative wavelengths and improve the predicted ability of models.

**Table 4:** Performance of different models in calibration and prediction for predicting color (ΔL*, Δa* and Δb*).

Model	Calibration						Prediction						Bands^a^
	ΔL*		Δa*		Δb*		ΔL*		Δa*		Δb*		
	*r_c_*	*RMSEC*	*r_c_*	*RMSEC*	*r_c_*	*RMSEC*	*r_p_*	*RMSEP*	*r_p_*	*RMSEP*	*r_p_*	*RMSEP*	
LS-SVM	0.972	0.741	0.973	0.463	0.967	1.354	0.931	1.164	0.973	1.051	0.933	1.936	496/496/496
PCR	0.908	1.302	0.799	1.169	0.915	2.134	0.927	1.188	0.762	1.375	0.921	2.083	496/496/496
CARS-LS-SVM	0.980	0.616	0.968	0.492	0.965	1.381	0.920	1.251	0.842	1.164	0.944	1.762	48/34/26
CARS-PLS	0.955	0.930	0.874	0.944	0.944	1.752	0.924	1.219	0.806	1.257	0.925	2.036	48/34/26
CARS-PCR	0.904	1.333	0.873	0.948	0.934	1.901	0.922	1.223	0.812	1.241	0.915	2.159	48/34/26
CARS-MLR	0.963	0.838	0.907	0.820	0.951	1.648	0.931	1.160	0.807	1.256	0.931	1.958	48/34/26
SPA-LS-SVM	0.933	1.116	0.893	0.877	0.916	2.133	0.929	1.178	0.849	1.146	0.917	2.142	7/6/11
SPA-PLS	0.914	1.263	0.809	1.143	0.904	2.265	0.925	1.205	0.746	1.415	0.907	2.258	7/6/11
SPA-PCR	0.911	1.285	0.669	1.446	0.891	2.417	0.924	1.219	0.754	1.397	0.904	2.287	7/6/11
SPA-MLR	0.917	1.247	0.810	1.140	0.906	2.244	0.926	1.201	0.754	1.397	0.908	2.254	7/6/11

a Number of input bands of ΔL*/Number of input bands of **Δ**a*/Number of input bands of **Δ**b*.

### Classification of different samples

The correct classification rates (CCRs) of samples at five different drying periods based on LS-SVM model were shown in Table 5. The results covered from 89.29% to 100% in the calibration set and from 71.43% to 100% in the prediction set, respectively. The total CCRs were 96.43% in the calibration set and 85.71% in the prediction set, respectively. From the results, it can be seen that type (6 min) and type (8 min) were identified badly (both were 71.43%), other three types were classified excellently with high values of CCRs (from 92.86% to 100%). There were three samples in type (6 min) were identified as type (8 min), and four ones in type (8 min) were identified as type (6 min). This might because of the short interval of drying time, which caused tiny change of color parameters of tea leaves. However, the total result was acceptable which demonstrated that hyperspectral imaging technique combined with LS-SVM model could also be used to classify tea leaves at different drying periods.

**Table 5:** Correct classification rates based on LS-SVM.

	Calibration set			Prediction set		
Types	No.	Missed	CCR^a^/%	No.	Missed	CCR^a^/%
0 min	28	0	100	14	1	92.86
4 min	28	0	100	14	0	100
6 min	28	2	92.86	14	4	71.43
8 min	28	3	89.29	14	4	71.43
10 min	28	0	100	14	1	92.86
Total	140	5	96.43	70	10	85.71

a Correct classification rates.

## Conclusions

This study was carried out to evaluate the feasibility of using visible and near infrared hyperspectral imaging technique, which covers the spectral wavelengths of 380 to 1030 nm, to determine color parameters of tea leaves during different drying periods. Two wavelengths selection methods including CARS and SPA were used to select effective wavelengths. Four different models (LS-SVM, PLS, PCR and MLR) were used to predict color values. Each wavelength selection method and each model obtained a good result. Among all models, the values of *r_p_* ranged from 0.902 to 0.931 for ΔL*, from 0.618 to 0.973 for Δa* and from 0.904 to 0.944 for Δb*, respectively. Based on the selected wavelengths, multispectral imaging system could be designed for nondestructive quality inspection during tea processing industry. Moreover, the CCRs of tea leaves at five different drying periods based on LS-SVM model covered from 89.29% to 100% in calibration set and from 71.43% to 100% in prediction set, respectively. The result demonstrates that this technique could to be used as an objective and nondestructive method to determine color parameters of tea leaves and classify samples during different drying periods. This is the first time that the visible and near infrared hyperspectral imaging technique was applied in the color determination of tea leaves at different drying periods. This technique can also be considered to determine some other chemical components which are also very important for tea's quality.

However, this study was a preliminary work. In further studies, more samples with different drying time should be selected to build more accurate and robust model. More effective wavelengths with higher accuracy and fewer variables should also be considered.
